# Impact of kidney function and kidney volume on intracranial aneurysms in patients with autosomal dominant polycystic kidney disease

**DOI:** 10.1038/s41598-022-22884-9

**Published:** 2022-10-27

**Authors:** Hiroshi Kataoka, Hiroyuki Akagawa, Rie Yoshida, Naomi Iwasa, Yusuke Ushio, Taro Akihisa, Masayo Sato, Shun Manabe, Shiho Makabe, Keiko Kawachi, Junichi Hoshino, Ken Tsuchiya, Kosaku Nitta, Toshio Mochizuki

**Affiliations:** 1 Department of Nephrology, Tokyo Women’s Medical University, 8-1 Kawada-Cho, Shinjuku-Ku, Tokyo, 162-8666 Japan; 2 Clinical Research Division for Polycystic Kidney Disease, Department of Nephrology, Tokyo Women’s Medical University, Tokyo, Japan; 3 Tokyo Women’s Medical University Institute for Integrated Medical Sciences (TIIMS), Tokyo, Japan; 4 Department of Blood Purification, Tokyo Women’s Medical University, Tokyo, Japan

**Keywords:** Diseases, Nephrology, Neurology

## Abstract

Presently, only personal or family history of intracranial aneurysm/subarachnoid hemorrhage (IA/SAH) has been established as a risk factor for IA in autosomal dominant polycystic kidney disease (ADPKD). This study aimed to verify the association between kidney function/volume and IAs in patients with ADPKD. This study included 519 patients with ADPKD. At baseline IA screening, the median age and estimated glomerular filtration rate were 44 years and 54.5 mL/min/1.73 m^2^, respectively. Family IA/SAH history was confirmed in 18.1% of the patients, and 54.3% of the patients had hypertension. The IA point prevalence was 12.5%. During clinical follow up of 3104 patient-years, de novo IA was detected in 29 patients (0.93% patient-years). The IA period prevalence was 18.1% (median age, 60 years). Multivariable logistic regression demonstrated that total kidney volume (TKV) ≥ 1000 mL (odds ratio [OR] = 2.81), height-adjusted TKV ≥ 500 mL (OR = 2.81), Mayo imaging classification Class 1D–1E (OR = 2.52), and chronic kidney disease stages 3–5 (OR = 2.31) were significantly associated with IA formation. IAs in patients with ADPKD may be associated not only with general risk factors for IAs but also with declining kidney function and increased KV. Kidney disease progression may contribute to effective IA screening and treatment planning in patients with ADPKD.

## Introduction

The prevalence of intracranial aneurysm (IA) is higher in patients with autosomal dominant polycystic kidney disease (ADPKD) (9–23%)^1–6^ than in the general population (2–4%)^7,8^. Female sex, increased age, subarachnoid hemorrhage (SAH) history, and ADPKD^9,10^ have been identified as IA risk factors in the general population; nonetheless, to date, only personal or family IA/SAH history has been established as a risk factor for IA in patients with ADPKD^11^. Until recently, universal IA screening in patients with ADPKD was not recommended by nephrologists^12,13^. Therefore, in regions where targeted screening is performed, understanding the overall IA picture in patients with ADPKD can be difficult because it is strongly affected by selection bias. In contrast, universal IA screening for patients with ADPKD has generally been conducted and recommended in Japan^14,15^. ADPKD itself is a risk factor for IA formation in the general population^9,10^, and mutation in *PKD1* and *PKD2* and its genotypes affect total kidney volume (TKV)^16^, kidney disease severity^17–21^, and IA^22,23^, which suggests that factors associated with ADPKD, including mutations in *PKD1* and *PKD2*, TKV, and kidney function are also probable risk factors for IA in patients with ADPKD. We hypothesized that owing to its genetic etiology, increased KV and declining kidney function would be responsible to a greater degree for IA formation in patients with ADPKD compared to general IA risk factors (such as female sex, increased age, and hypertension). In this study, we analyzed the IA/SAH point and period prevalence, novel IA/SAH incidence, and risk factors for IA in a relatively large cohort of patients with ADPKD at a single Japanese institution.

## Results

### Patient characteristics

The entire cohort’s patient characteristics are summarized in Tables 1 and S1. During baseline IA screening, the median age, estimated glomerular filtration rate (eGFR), and TKV were 44 years, 54.5 mL/min/1.73 m^2^, and 1054.3 mL, respectively. Family IA/SAH history was confirmed in 94 (18.1%) patients, and 282 (54.3%) patients had hypertension. The IA prevalence and SAH point prevalence among the 519 patients with ADPKD at baseline screening were 12.5% (65 patients) and 3.1% (16 patients), respectively. During a clinical follow up of 3104 patient-years in these 519 patients, de novo IAs were detected in 29 patients (0.93% [95% confidence interval (CI), 0.62–1.34%] patient-years for saccular IAs) and three patients had IA rupture (0.10% [95% CI, 0.02–0.28%] patient-years). Accordingly, at the time of the last follow up (median, 50 years), the IA and SAH period prevalence among the 519 patients with ADPKD was 18.1% (94 patients) and 3.7% (19 patients), respectively.

**Table 1:** Patients’ characteristics according to the presence or absence of intracranial aneurysm.

Variables	Entire cohort n = 519	Patients with IAs n = 94	Patients without IAs n = 425	*P* value
**Clinical findings**				
Age at the time of first screening (years)	44 (12–86) [519]	47.5 (18–77)	43 (12–86)	0.0052*
Follow-up duration (years)	5.98 ± 4.78 [519]	5.92 ± 4.86	5.99 ± 4.76	0.8971
Age at the time of last follow-up (years)	50 (13–88) [519]	53 (19–83)	49 (13–88)	0.0089*
Female sex, n (%)	284 (54.7) [519]	55 (58.5)	229 (53.9)	0.4146
Family history of IA or SAH, n (%)	94 (18.1) [519]	27 (28.7)	67 (15.8)	0.0032*
**Brain findings**				
Total IA, n (%)	94 (18.1) [519]	94 (100.0)	0 (0.0)	NA
Baseline IA, n (%)	65 (12.5) [519]	65 (69.2)	0 (0.0)	NA
IA during follow-up, n (%)	29 (5.6) [519]	29 (30.9)	0 (0.0)	NA
Total SAH, n (%)	19 (3.7) [519]	19 (20.2)	0 (0.0)	NA
Baseline SAH, n (%)	16 (3.1) [519]	16 (17.0)	0 (0.0)	NA
SAH during follow-up, n (%)	3 (0.6) [519]	3 (3.2)	0 (0.0)	NA
**Kidney findings**				
eGFR (mL/min/1.73 m^2^)	54.5 (3.0–150.1) [519]	35.2 (3.1–125.8)	59.0 (3.0–150.1)	< 0.0001*
Blood urea nitrogen (mg/dL)	18.4 (2.9–109.4) [518]	23.7 (9.9–91.3)	17.1 (2.9–109.4)	< 0.0001*
CKD 3–5, n (%)	287 (55.3) [519]	69 (73.4)	218 (51.3)	< 0.0001*
U-Prot (g/g･Cre)	0.07 (0.00–10.32) [517]	0.15 (0.00–7.14)	0.06 (0.00–10.32)	0.0030*
TKV (mL)	1054.3 (196.4–8695.2) [449]	1459.1 (431.2–8695.2)	947.9 (196.4–5920.4)	< 0.0001*
TKV ≥ 1000 mL	237 (52.8) [449]	59 (73.8)	178 (48.2)	< 0.0001*
htTKV (mL)	652.4 (129.2–5023.2) [436]	963.8 (288.5–5023.2)	599.6 (129.2–3482.6)	< 0.0001*
htTKV ≥ 500 mL	271 (62.2) [449]	64 (81.1)	207 (59.7)	< 0.0001*
Mayo imaging classification Class 1D–1E, n (%)	116 (27.0) [429]	30 (38.0)	86 (24.6)	0.0154*
**Comorbidities**				
Hypertension, n (%)	282 (54.3) [519]	66 (70.2)	216 (50.8)	0.0006*
Hyperuricemia, n (%)	157 (30.3) [519]	42 (44.7)	115 (27.1)	0.0008*
Low HDL cholesterol, n (%)	73 (14.1) [519]	21 (22.3)	52 (12.2)	0.0108*

*P < 0.05. Continuous values are expressed as the mean ± standard deviation or median (range). Discrete data are expressed as n (%). Values for numbers of subjects are shown in [].

*IA* Intracranial aneurysm, *n* Number, *%* Percentage, *SAH* Subarachnoid hemorrhage, *CKD* Chronic kidney disease, *eGFR* Estimated glomerular filtration rate, *U-Prot* Urinary protein excretion, *TKV* Total kidney volume, *htTKV* Height-adjusted TKV, *HDL* High-density lipoprotein.

Comparative analyses of the data of patients with and those without IA revealed that the frequency of family IA/SAH history was higher among patients with IAs than among those without (28.7% vs. 15.8%, *P* = 0.0032). Baseline hypertension (70.2% for patients with IA vs. 50.8% for those without, *P* = 0.0006) was more frequent and the patients were older on average (47.5 vs. 43 years, *P* = 0.0052) in the aneurysm group. Regarding kidney findings during baseline IA screening, the median eGFR was significantly lower in patients with IA (35.2 mL/min/1.73 m^2^) than in those without (59.0 mL/min/1.73 m^2^; *P* < 0.0001), and the median TKV was significantly higher in patients with IA than in those without (1459.0 mL with IA vs. 947.9 mL without IA; *P* < 0.0001). The proportions of patients with chronic kidney disease (CKD) stages 3–5 (73.4% with IA vs. 51.3% without IA, *P* < 0.0001) and those with Mayo imaging classification Class 1D–1E (38.0% with IA vs. 24.6% without IA, *P* = 0.0154) were higher in the aneurysm group.

### Characteristics of patients with IAs

IA-associated patient and aneurysm characteristics are shown in Table 2. Ninety-four patients were diagnosed at a median age of 46 (range, 18–81) years, and 24 (25.5% of patients with IA) presented with multiple aneurysms. All IAs were small (maximum IA diameter < 10 mm), with a median maximum IA diameter of 3.5 (range, 2.0–8.3) mm. The most frequent IA site was the middle cerebral artery in the anterior circulation. Nineteen patients (20.2%) experienced aneurysm rupture, and the median age at the time of rupture was 39 (range, 29–71) years. Treatment included neurosurgical procedures with IA clipping or coiling in 51 (54.3%) patients (clipping, 42 patients; coiling, nine patients).

**Table 2:** Characteristics of aneurysms and patients with IAs (n = 94).

Patient characteristics associated with IA/SAH	
**IA**	
Age at diagnosis of IAs (years)	46 (18–81)
Multiple IAs at baseline screening, n (%)	14 (14.9)
Multiple IAs during follow-up, n (%)	10 (10.6)
Numbers of IAs	1 (1–6)
Size of the largest aneurysm: maximum IA diameter (mm)	3.5 (2.0–8.3)
Treatments, n (%)	51 (54.3)
Clipping, n (%)	42 (44.7)
Coiling, n (%)	9 (9.6)
**SAH**	
SAH, n (%)	19 (20.2)
Age at SAH (years)	39 (29–71)

Location of the IAs	
**Anterior circulation, n (%)**	**73 (66.4) {110}**
A-com, n (%)	10 (9.1) {110}
ACA, n (%)	8 (7.3) {110}
MCA, n (%)	34 (30.9) {110}
ICA, n (%)	21 (19.1) {110}
**Posterior communicating artery, n (%)**	**16 (14.5) {110}**
IC-PC, n (%)	16 (14.5) {110}
**Posterior circulation, n (%)**	**21 (19.1) {110}**
BA, n (%)	9 (8.2) {110}
VA, n (%)	7 (6.4) {110}
PICA, n (%)	3 (2.7) {110}
PCA, n (%)	2 (1.8) {110}

Continuous values are expressed as the median (range). Discrete data are expressed as n (%). Values of numbers of IAs where the location were identified are shown in {}.

*IA* Intracranial aneurysm, *SAH* Subarachnoid hemorrhage, *n* Number, *%* Percentage, *A-com* Anterior communicating artery, *ACA* Anterior cerebral artery, *MCA* Middle cerebral artery, *ICA* Internal carotid artery, *IC-PC* Internal carotid-posterior communicating artery, *BA* Basilar artery, *VA* Vertebral artery, *PICA* Posterior inferior cerebellar artery, *PCA* Posterior cerebral artery.

Total numbers and % of aneurysms for each cerebral circulation are shown in bold.

### IA/SAH and risk-factor associations

**Figure 1:**
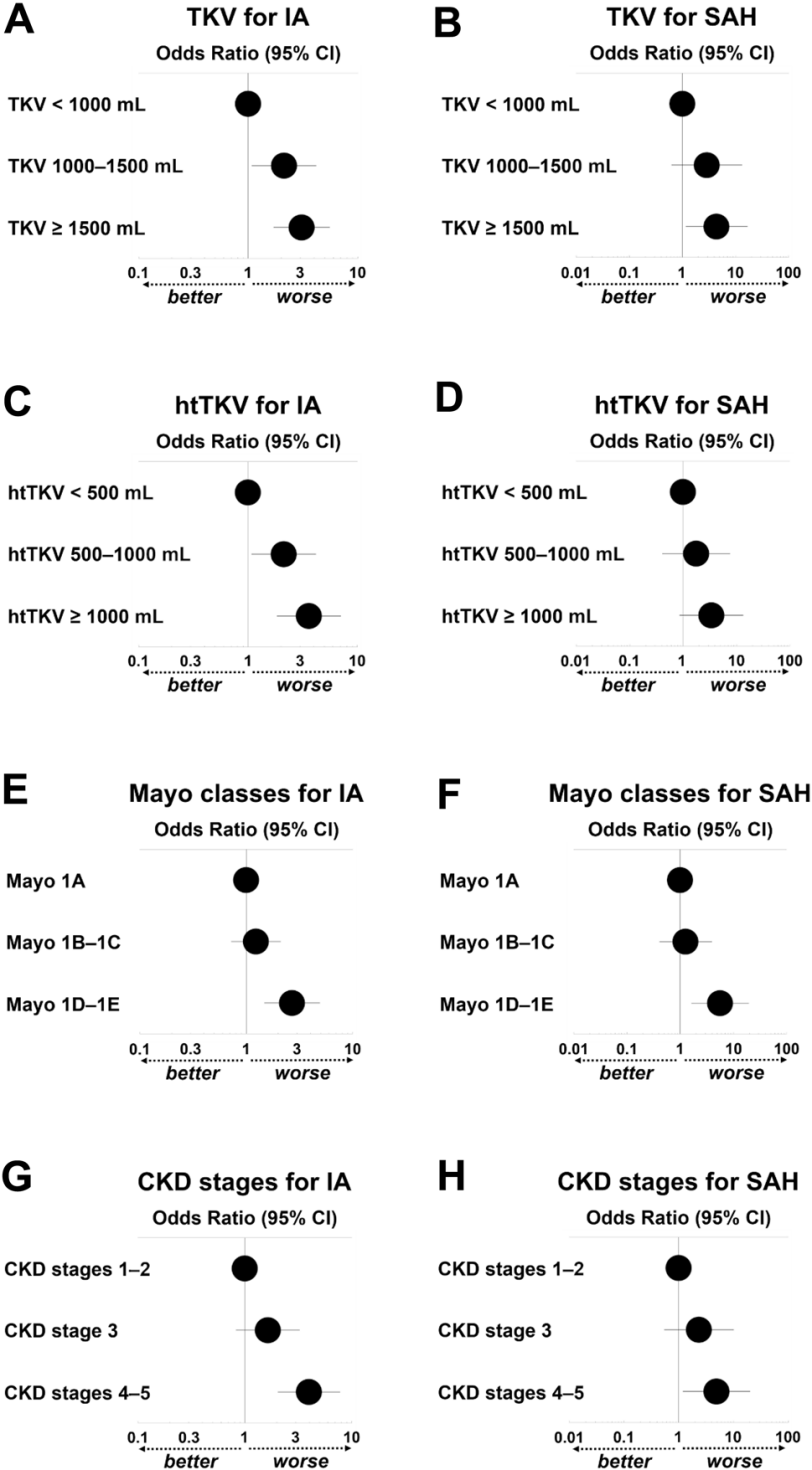
Odds ratios for IA/SAH derived from the age-adjusted logistic regression analyses (**A**–**H**). The circles represent odds ratios, and the bars represent 95% CI for the association with IA/SAH (derived from Table S2). (**A**) TKV for IA. (**B**) TKV for SAH. (**C**) htTKV for IA. (**D**) htTKV for SAH. (**E**) Mayo imaging classification for IA. (**F**) Mayo imaging classification for SAH. (**G**) CKD stages for IA. (**H**) CKD stages for SAH. Abbreviations: IA, intracranial aneurysm, SAH, subarachnoid hemorrhage, CI, confidence interval, TKV, total kidney volume, htTKV, height-adjusted TKV; Mayo, Mayo imaging classification; CKD, chronic kidney disease.

First, we investigated the relationship between the progression of ADPKD-related factors and IA/SAH. Thus, risk factors for IA in the general population as well as ADPKD-related factors, including TKV (< 1000 mL, 1000–1500 mL, and ≥ 1500 mL), height-adjusted TKV (htTKV) (< 500 mL, 500–1000 mL, and ≥ 1000 mL), Mayo imaging classification (Classes 1A, 1B–1C, and 1D–1E), and CKD stages (1–2, 3, and 4–5), were examined using age-adjusted logistic regression analyses (Table S2, Fig. 1). As the TKV (Fig. 1A–B) and htTKV increased (Fig. 1C–D), and as the Mayo class (Fig. 1E–F) and CKD stage advanced (Fig. 1G–H), odds ratios (ORs) for IA/SAH increased.

Multivariable logistic regression analyses using the general risk factors for IA, as well as TKV, htTKV, Mayo classification, or CKD stage, were performed for IA formation (Table 3 and Table S3, upper part). In the model using general risk factors for IA, hypertension (OR = 2.58, *P* = 0.0006) and family history of IA/SAH (OR = 2.41, *P* = 0.0013) were significantly associated with IA formation (McFadden’s pseudo-*R*^2^ = 0.06, the area under the receiver operating characteristic curve [AUC] = 0.66). Adding TKV to the model of general risk factors increased the AUC to 0.72 and the pseudo-*R*^2^ value to 0.11. The TKV (100 mL increase; OR = 1.05, *P* < 0.0001) was significantly associated with IA formation, indicating that adding TKV improved the model’s discriminatory ability and goodness-of-fit to predict IAs. Similarly, adding htTKV, Mayo 1D–1E, and eGFR to the model of general risk factors also increased the AUC (0.72, 0.71, and 0.70, respectively) and pseudo-*R*^2^ value (0.11, 0.10, and 0.08, respectively). The htTKV (100-mL increase; OR = 1.09, *P* < 0.0001), Mayo 1D–1E (OR = 2.90, *P* = 0.0012), and eGFR (10 mL/min/1.73 m^2^ decrease; OR = 1.21, *P* = 0.0004) were significantly associated with IA diagnosis. Therefore, adding htTKV, Mayo 1D–1E, and eGFR improved the model’s discriminatory ability and goodness-of-fit to predict IAs. Similarly, multivariable logistic regression analyses based on binary data confirmed ADPKD-related factors, including TKV ≥ 1000 mL (OR = 2.81, *P* = 0.0006), htTKV ≥ 500 mL (OR = 2.81, *P* = 0.0018), Mayo 1D–1E (OR = 2.52, *P* = 0.0037), and CKD stages 3–5 (OR = 2.31, *P* = 0.0062) to be significantly associated with IA formation (Tables 3, S3, lower part; Fig. S1).

**Table 3:** Multivariable logistic regression analyses for correlations between a diagnosis of intracranial aneurysm and risk factors.

Variables	General risk factors (*n* = 519)		General risk factors with TKV (*n* = 449)		General RISK FACTORS WITH eGFR (*n* = 519)	
	(AICc = 472.8, pseudo-*R*^2^ = 0.06, AUC = 0.66)		(AICc = 385.9, pseudo-*R*^2^ = 0.11, AUC = 0.72)		(AICc = 461.8, pseudo-*R*^2^ = 0.08, AUC = 0.70)	
	Odds Ratio (95% CI)	*P* value	Odds Ratio (95% CI)	*P* value	Odds Ratio (95% CI)	*P* value
Female (vs. male)	1.58 (0.97–2.58)	0.0677	1.93 (1.08–3.46)	0.0270*	1.91 (1.15–3.17)	0.0128*
Hypertension	2.58 (1.50–4.43)	0.0006*	2.15 (1.16–3.99)	0.0154*	2.05 (1.17–3.57)	0.0116*
Family history of IA or SAH	2.41 (1.41–4.14)	0.0013*	2.91 (1.59–5.31)	0.0005*	2.46 (1.42–4.25)	0.0013*
Age (10-year increments)	1.14 (0.96–1.36)	0.1224	1.16 (0.95–1.42)	0.1496	0.91 (0.73–1.13)	0.3967
TKV (100 mL increase)	NA	NA	1.05 (1.03–1.08)	< 0.0001*	NA	NA
eGFR (10 mL/min/1.73 m^2^ decrease)	NA	NA	NA	NA	1.21 (1.09–1.35)	0.0004*

Variables	General risk factors (*n* = 519)		General risk factors with TKV ≥ 1000 mL (*n* = 449)		General risk factors with CKD3–5 (*n* = 519)	
	(AICc = 473.5, pseudo-*R*^2^ = 0.06, AUC = 0.66)		(AICc = 391.9, pseudo-*R*^2^ = 0.10, AUC = 0.72)		(AICc = 467.8, pseudo-*R*^2^ = 0.07, AUC = 0.69)	
	Odds ratio (95% CI)	*P* value	Odds ratio (95% CI)	*P* value	Odds ratio (95% CI)	*P* value
Female (vs. male)	1.55 (0.95–2.54)	0.0786	1.64 (0.94–2.86)	0.0786	1.76 (1.06–2.92)	0.0278*
Hypertension	2.70 (1.58–4.60)	0.0003*	2.30 (1.25–4.22)	0.0071*	2.25 (1.30–3.89)	0.0037*
Family history of IA or SAH	2.45 (1.43–4.19)	0.0011*	3.04 (1.68–5.49)	0.0002*	2.35 (1.36–4.05)	0.0021*
Age ≥ 50 years	1.38 (0.85–2.33)	0.1889	1.27 (0.74–2.18)	0.3769	0.97 (0.57–1.66)	0.9178
TKV ≥ 1000 mL	NA	NA	2.81 (1.56–5.09)	0.0006*	NA	NA
CKD 3–5	NA	NA	NA	NA	2.31 (1.27–4.19)	0.0062*

*P < 0.05. Variables of general risk factors for intracranial aneurysms and TKV, and eGFR were included in the multivariable models.

*n* Number, *TKV* Total kidney volume, *eGFR* Estimated glomerular filtration rate, *CI* Confidence interval, *P* Calculated probability, *AICc* Small-sample corrected akaike information criterion, *pseudo-R*^*2*^ McFadden’s pseudo-*R*-squared, *AUC* Area under the receiver operating characteristic curve, *IA* Intracranial aneurysm, *SAH* Subarachnoid hemorrhage, *NA* Not applicable.

**Figure 2:**
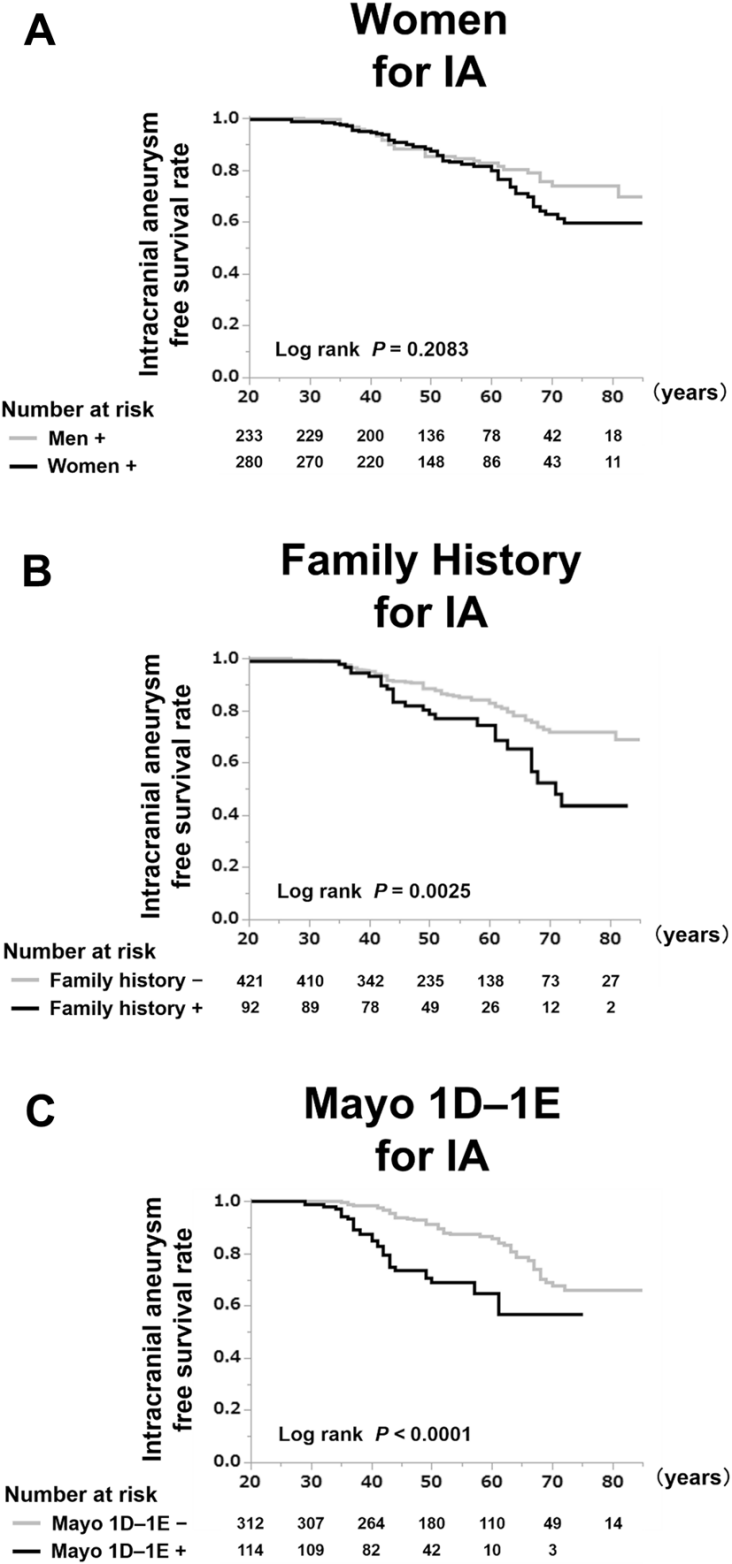
IA diagnosis-free survival rate of patients with autosomal dominant polycystic kidney disease (ADPKD) stratified by sex, family history, and Mayo 1D–1E. (**A**) The IA diagnosis-free survival rates of men and women (log-rank, *P* = 0.2083). (**B**) The IA diagnosis-free survival rates of patients with ADPKD with family history (log-rank, *P* = 0.0025). (**C**) The IA diagnosis-free survival rates of patients with ADPKD with Mayo 1D–1E (log-rank, *P* < 0.0001). A Kaplan–Meier curve based on the age when IAs were confirmed by clinicians. The number of patients at risk of progression to IA diagnosis at each time point is mentioned below the figures. Abbreviations: IA, intracranial aneurysm, family history, family history of intracranial aneurysm or subarachnoid hemorrhage, Mayo 1D–1E, Mayo imaging classification 1D–1E.

In the age-specific Kaplan–Meier model, which was based on the age at IA confirmation (Fig. 2), the IA-free survival rates did not differ between men and women, especially before the age of 50 years (Fig. 2A). In contrast, the IA-free survival rates in patients with a family history of IA/SAH and Mayo 1D–1E were significantly lower than those in patients without a family history of IA/SAH and without Mayo 1D–1E, even before the age of 50 years (Fig. 2B–C; log-rank, *P* = 0.00025, log-rank, *P* < 0.0001, respectively).

## Discussion

ADPKD, the most common progressive hereditary kidney disease^20,24^, causes cyst formation in the kidneys, leading to various extrarenal complications, including liver cysts^25^, hypertension, and IAs. Generally, patients with ADPKD carry a germline mutation in one allele of either *PKD1* or *PKD2*^17–19^. This study revealed multiple IA risk factors in patients with ADPKD. To the best of our knowledge, this was the first study to confirm the association between IA/SAH and kidney function in patients with ADPKD. Multivariable analyses demonstrated that a family history of IA/SAH, hypertension, TKV/htTKV increase (or TKV ≥ 1000 mL/htTKV ≥ 500 mL), Mayo 1D–1E, and decrease in eGFR (or CKD stages 3–5) were significantly associated with IA diagnosis.

Thus far, as IA prevalence and the risk of small IA ruptures have been considered to be low, indications for IA screening in patients with ADPKD have been limited to those with a family history of IA/SAH, previous IA rupture, a high-risk profession (e.g., airline pilots), and anxiety despite adequate information^12,13^. However, the considerable variability in IA prevalence (9–23%) in patients with ADPKD^1–3^ indicates the need to further examine this issue. First, it is difficult to accurately determine the IA prevalence and risk levels in patients with ADPKD who have undergone targeted screening, which is theoretically prone to selection bias. Furthermore, accurate information regarding family history of IA/SAH is indeterminable without universal IA screening. Therefore, research reports from regions where universal IA screenings are conducted may be useful.

Recently reported risk factors for IA in patients with ADPKD based on the results of multivariable analyses include: family history of hemorrhagic stroke or IA (355 patients in China)^1^; TKV (265 patients in Japan)^5^; age, female sex, intracranial arterial dolichoectasia, and mitral inflow deceleration time for a limited subgroup of high-risk aneurysms (926 patients in Korea)^6^; and age > 45 years (83 patients in Poland)^3^. However, no reports have confirmed the association between kidney function and IA/SAH in patients with ADPKD. That said, Yoshida et al.^5^ found a significant association between TKV and IA/SAH in patients with ADPKD, but not kidney function expressed as eGFR (*P* = 0.07), which may have been influenced by their smaller study scale (265 patients) than ours (519 patients). In this study, age, female sex, hypertension, TKV, htTKV, Mayo imaging classification of ADPKD, and kidney function showed independent associations with IAs in patients with ADPKD, and our cohort’s large size probably contributed to the results. Hypertension, TKV, htTKV, Mayo imaging classification of ADPKD, and kidney function showed independent associations with IA in all evaluated models, while associations of age or female sex with IA were model dependent. As adding ADPKD-related risk factors to general IA risk factors improved the model’s discriminatory ability and goodness-of-fit to predict IAs, ADPKD-related risk factors such as kidney function/volume could be potentially more important IA risk factors in patients with ADPKD than are general IA risk factors.

We recently conducted a detailed analysis of genetic factors, including mutation types, in patients with ADPKD and IA and found an association between mutation type such as splicing/frameshift mutations and IAs in patients with ADPKD^23^. As splicing/frameshift mutations are significantly associated with large TKV^16^, advanced classes of the Mayo imaging classification^16^, and a poor renal prognosis^20^, patients with ADPKD with progressive kidney dysfunction or increasing TKV could have splicing/frameshift mutations and consequently be at risk of IA formation. Interestingly, though splicing mutations were significantly associated with IA formation not only in the entire cohort but also in patients with CKD stages 1–3, substitutions showed significant interactions with CKD stages 4–5 and showed a significant association with IA formation only in the sub-cohorts of patients with CKD stages 4–5^23^. This result regarding ADPKD with substitutions suggested the possibility that kidney function itself affects IA formation. Findings in the baseline IA screening of the present study show that eGFR was significantly lower in patients with IA (35.2 mL/min/1.73 m^2^) than in those without (59.0 mL/min/1.73 m^2^) and two recent reports on high prevalence of IA in ADPKD patients with end-stage kidney disease (14.9% of 154 patients mean-aged 51.2 years in Korea^26^, 22.7% of 66 patients median-aged 60.9 years in Poland^27^) may suggest such an association.

Currently, no consensus has been formed for universal screening of IA in patients with ADPKD^12,13,28^. However, regardless of whether universal^11,28,29^ or targeted screenings^12,30^ are used, it is important to clarify the risk factors for IAs^13^ and for patients with ADPKD to be informed of their risk of IA formation. As genotypes and mutation types of ADPKD affect total kidney volume (TKV)^16^, kidney disease severity^17–21^, and IA^22,23^, genetic tests may contribute to personalized medicine/precision medicine for patients with ADPKD^16,20,23^. Reducing costs of genotyping could make it more accessible for genetic tests, and genotypes and mutation types of ADPKD would be included in the indication criteria for IA screening in future^23^. However, kidney dysfunction itself has an association with IA formation. Markers of kidney cystic progression and kidney function decline would be a widely applicable criterion for the time being and could be used for screening.

This study has some limitations. First, it was an observational study, and a causal relationship could not be proven based on our observations. TKV values were calculated using an ellipsoid formula instead of manual planimetry. No correction was made for the multiplicity of testing, given the exploratory nature of the study. Furthermore, although this study’s strength was its ability to evaluate the vast amount of data stored in the electronic medical records, smoking history was not evaluated.

In conclusion, in this study, IA in patients with ADPKD was associated with general risk factors for IA and with declining kidney function and increased KV. The factors observed to be associated with IA in our study may contribute to effective IA screening and treatment planning in patients with ADPKD.

## Methods

### Study design

**Figure 3:**
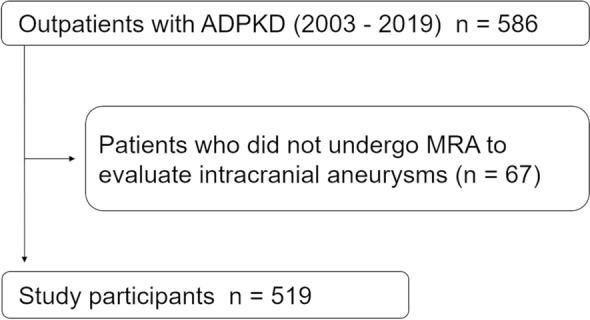
Patient selection flow chart. Abbreviations: ADPKD, autosomal dominant polycystic kidney disease, MRA, magnetic resonance angiography.

We reviewed the records of 586 outpatients with ADPKD who visited the Kidney Center, Tokyo Women’s Medical University Hospital (Tokyo, Japan), between July 2003 and July 2019. Our facility has been conducting universal IA screening since the 2000s. Universal screenings have been conducted every 3–5 years in patients with ADPKD; if IAs are detected, follow-up magnetic resonance angiography (MRA) is performed every 1–2 years^14,15,31^. After excluding 67 patients who did not undergo an MRA intracranial examination, 519 patients were included in the study (Fig. 3; screening ratio, 88.6%). All procedures were approved by the Research Ethics Committee of Tokyo Women’s Medical University (approval number, 5118) and were in accordance with the 1964 Declaration of Helsinki and its later amendments or comparable ethical standards. Passive informed consent (in the form of opt-out) was obtained from the patients. All data were analyzed anonymously. The participants were followed up until October 31, 2020.

### Covariate assessments

During a regular outpatient clinic visit, anthropometric and physical examinations were conducted, including blood-pressure, height, and weight assessments. All biochemical analyses were performed on samples obtained from patients after overnight fasting. The eGFR for Japanese patients was calculated using a previously described formula^32^. IA and TKV assessments and comorbidity definitions are presented in Supplementary Methods.

### Outcome evaluations

The primary endpoint was IA formation/SAH confirmed with universal MRA screening or based on the medical records. The risk of de novo IA formation was calculated as de novo saccular aneurysm formation based on MRA screening divided by the mean follow up in years. Similarly, SAH risk per patient-year was calculated as SAH incidence in the entire population divided by the mean follow up in years.

### Statistical analyses

Continuous variables are expressed as means ± standard deviations or medians (range), while discrete variables are expressed as percentages. The Mann–Whitney U-test or unpaired t-test was performed for continuous variables after assessing data normality. The chi-square or Fisher’s exact test was used to analyze categorical variables. Logistic regression analyses were performed to determine factors associated with IA diagnosis. Multivariable logistic regression models were used to estimate the associated risk of IA diagnosis. The variables of interest and general risk factors for outcomes based on existing knowledge were included in the multivariable model. Since KV, Mayo imaging classification, and kidney function demonstrated strong correlations^24,33^, we constructed four multivariate logistic models separately, considering multicollinearity^34^. Standard methods were used to estimate the sample size for multivariable logistic regression with ≥ 5 outcomes required for each independent variable^35,36^. Discriminatory ability was measured using AUCs. Goodness-of-fit was assessed using McFadden’s pseudo-*R*-squared (pseudo-*R*^2^)^37^ and the small-sample-corrected Akaike’s information criterion^38^. The time from birth to IA diagnosis was computed using the Kaplan–Meier method and evaluated using the log-rank test. Considering the study’s exploratory nature, we did not adjust for multiplicity. Statistical significance at 5% was considered a marker for potential further investigation. All statistical tests were two-tailed, and all statistical analyses were performed using JMP Pro software (version 15.0.0; SAS Institute, Cary, NC, USA).

## Supplementary Information

Supplementary Information.

## Data Availability

The datasets generated and/or analysed during the current study are not publicly available due to containing information that could compromise the privacy of research participants but are available from the corresponding author on reasonable request.
