# Supplementary material for: Impact of kidney function and kidney volume on intracranial aneurysms in patients with autosomal dominant polycystic kidney disease
Source: Sci Rep. 2022 Oct 27;12:18056. doi: 10.1038/s41598-022-22884-9 (PMC9613770; doi:10.1038/s41598-022-22884-9)
Supplement: Supplementary file 1 — Supplementary Information. [file 41598_2022_22884_MOESM1_ESM.docx]

**Supporting information to “Impact of Kidney Function and Kidney Volume on Intracranial Aneurysms in Patients with Autosomal Dominant Polycystic Kidney Disease”**

This supplementary information provides further methodological detail and results for the main paper.

**Supporting information**

**Supplementary Methods**

- Intracranial Aneurysm Assessment
- Total Kidney Volume Assessment
- Comorbidity Definitions

**S1 Table.** Patients’ characteristics according to the presence or absence of intracranial aneurysm^*^

**S2 Table.** Age-adjusted logistic regression analyses of correlations between a diagnosis of intracranial aneurysm/subarachnoid hemorrhage and risk factors

**S3 Table.** Multivariable logistic regression analyses for correlations between a diagnosis of intracranial aneurysm and risk factors

**S1 Figure.** Odds ratios for a diagnosis of intracranial aneurysm from multivariable logistic regression analyses (A–D)

(A) General risk factors with total kidney volume ≥1000 mL for intracranial aneurysm.

(B) General risk factors with height-adjusted total kidney volume ≥500 mL for intracranial aneurysm.

(C) General risk factors with Mayo 1D–1E.

(D) General risk factors with chronic kidney disease stages 3–5.

^*^Remaining data after excluding the data for Table 1.

**Supplementary Methods: Detailed Methods**

***Intracranial Aneurysm Assessment***

All MRA examinations for IA were performed in clinical settings using 1.5- or 3.0-T MR scanners (Ingenia: Philips Medical Systems, Best, The Netherlands; Titan: Canon Medical Systems, Tokyo, Japan) to diagnose IA presence, size, number, and location. We evaluated the maximum IA diameter as an IA-size indicator. The maximum IA diameter was defined as the longest diameter of the largest IA in serial magnetic resonance imaging sections. The MRA images were examined under the following conditions: slice thickness 0.8–1.2 mm; slice interval: gapless spectrum; repetition time: 21 ms (3.0 T), 25 ms (1.5 T); echo time: 3.5 ms (3.0 T), 6.9 ms (1.5 T); field of view: 200 × 200 mm; and matrix: 256 × 256 (Ingenia 3.0 T), 332 × 183 (Ingenia 1.5 T), 272 × 256 (Titan 3.0 T), and 272 × 176 (Titan 1.5 T). Considering that the spatial resolutions of 1-T and 3-T scanners reach 1 mm [1] and 0.6 mm [2, 3], respectively, we investigated saccular cerebral aneurysms ≥ 2 mm in this study. IA evaluation was independently performed by two researchers (H.K. and D.Y.) based on radiologists’ clinical reports. Disagreement was resolved by consulting a neurosurgeon (H.A.) and researcher consensus.

***Total Kidney Volume Assessment***

Using MR or computed tomography images, TKV was estimated from the kidney’s maximum length, width, and depth using the formula for an ellipsoid: π/6 × length × width × depth.

***Comorbidity Definitions***

The impact of concomitant treatment and comorbidities was assessed [4]. Comorbidities were recorded as positive according to the following criteria: hypertension was defined as systolic BP ≥ 140 mmHg, diastolic BP ≥ 90 mmHg, or current antihypertensive-agent use; hypertriglyceridemia was defined as a serum triglyceride level ≥ 150 mg/dL or current anti-dyslipidemic agent use; low HDL-C was defined as a serum HDL-C level ≤ 40 mg/dL in men and ≤ 50 mg/dL in women or current anti-dyslipidemic agent use; high LDL-C was defined as a serum LDL-C level ≥ 140 mg/dL or current anti-dyslipidemic agent use; diabetes mellitus was defined as a glycated hemoglobin level ≥6.5%, diabetes mellitus diagnosis, or current antidiabetic-agent use.

1. Schellinger PD, Richter G, Kohrmann M, Dorfler A (2007) Noninvasive angiography (magnetic resonance and computed tomography) in the diagnosis of ischemic cerebrovascular disease. Techniques and clinical applications. Cerebrovasc Dis 24 Suppl 1:16-23

2. Bernstein MA, Huston J, 3rd, Lin C, Gibbs GF, Felmlee JP (2001) High-resolution intracranial and cervical MRA at 3.0T: technical considerations and initial experience. Magn Reson Med 46:955-962

3. Kaufmann TJ, Huston J, 3rd, Cloft HJ, Mandrekar J, Gray L, Bernstein MA, Atkinson JL, Kallmes DF (2010) A prospective trial of 3T and 1.5T time-of-flight and contrast-enhanced MR angiography in the follow-up of coiled intracranial aneurysms. AJNR Am J Neuroradiol 31:912-918

4. Ording AG, Sorensen HT (2013) Concepts of comorbidities, multiple morbidities, complications, and their clinical epidemiologic analogs. Clin Epidemiol 5:199-203

**S1 Table. Patients’ characteristics according to the presence or absence of intracranial aneurysm**

| **Variables** | **Entire cohort**  **n = 519** | **Patients with IAs n = 94** | **Patients without IAs n = 425** | ***P*–value** |
| --- | --- | --- | --- | --- |
| ***Clinical Findings*** |  |  |  |  |
| Mean blood pressure (mmHg) | 95.9 ± 10.2 [487] | 97.2 ± 8.3 | 95.6 ± 10.5 | 0.1869 |
| Body mass index (kg/m^2^) | 22.4 ± 3.2 [494] | 22.7 ± 3.5 | 22.3 ± 3.2 | 0.3762 |
| ***Kidney Findings*** |  |  |  |  |
| CKD1–2/ CKD3/ CKD4–5, n (%) | 232 (44.7) / 142 (27.4) / 145 (27.9) [519] | 25 (26.6) / 23 (24.5) / 46 (48.9) | 207 (48.7) / 119 (28.0) / 99 (23.3) | <0.0001* |
| Mayo imaging classification 1A/ Class1B/ Class1C/ Class1D/ Class1E/ Class2, n (%) | 46 (10.7) / 143 (33.3) / 124 (28.9) / 82 (19.1) / 34 (7.9) / 0 (0.0) [429] | 3 (3.8) / 26 (32.9) / 20 (25.3) / 23 (29.1) / 7 (8.9) / 0 (0.0) | 43 (12.3) / 117 (33.4) / 104 (29.7) / 59 (16.9) / 27 (7.7) / 0 (0.0) | 0.0415* |
| Mayo imaging classification Class1C–1E, n (%) | 240 (55.9) [429] | 50 (63.3) | 190 (54.3) | 0.1453 |
| ***Concomitant drugs*** |  |  |  |  |
| Antihypertensive agents, n (%) | 237 (45.7) [519] | 60 (63.8) | 177 (41.7) | <0.0001* |
| ARB and or ACEI | 196 (37.8) [519] | 49 (25.0) | 147 (34.6) | 0.0015* |
| Antidyslipidemic agents, n (%) | 35 (6.7) [519] | 11 (11.7) | 24 (5.7) | 0.0416* |
| Antihyperuricemic agents, n (%) | 80 (15.4) [519] | 24 (25.5) | 56 (13.2) | 0.0027* |
| Tolvaptan, n (%) | 7 (1.4) [519] | 1 (1.1) | 6 (1.4) | 1.0000 |
| ***Comorbidities*** |  |  |  |  |
| Hypertriglyceridemia, n (%) | 102 (19.7) [519] | 20 (21.3) | 82 (19.3) | 0.6616 |
| High LDL cholesterol, n (%) | 68 (13.1) [519] | 13 (13.8) | 55 (12.9) | 0.8173 |
| Diabetes mellitus, n (%) | 10 (1.9) [519] | 2 (2.1) | 8 (1.9) | 1.0000 |

**P* < 0.05. Remaining data after excerpting the data for Table 1. Continuous values are expressed as the mean ± standard deviation or median (range). Discrete data are expressed as n (%). Values for number of subjects are shown in []. Abbreviations: IA, intracranial aneurysm; n, number; %, percentage; CKD, chronic kidney disease; ARB, angiotensin Ⅱ receptor blocker; ACEI, angiotensin converting enzyme inhibitor; LDL low-density lipoprotein.

**S2 Table. Age-adjusted logistic regression analyses of correlations between a diagnosis of intracranial aneurysm/subarachnoid hemorrhage and risk factors**

|  | **IA** |  | **SAH** |  |
| --- | --- | --- | --- | --- |
| **Variables** | **Odds Ratio**  **(95% CI)** | ***P*-**  **Value** | **Odds Ratio**  **(95% CI)** | ***P*-**  **Value** |
| Women (vs. men) | 1.28 (0.81–2.02) | 0.2942 | 1.20 (0.47–3.05) | 0.7025 |
| Hypertension | 2.02 (1.22–3.34) | 0.0060* | 1.65 (0.59–4.60) | 0.3401 |
| Family history of IA or SAH | 2.22 (1.32–3.75) | 0.0028* | 2.83 (1.08–7.40) | 0.0453* |
|  |  |  |  |  |
| TKV < 1000 mL | 1 (reference) |  | 1 (reference) |  |
| TKV 1000–1500 mL | 2.18 (1.11–4.31) | 0.0242* | 2.88 (0.62–13.36) | 0.1759 |
| TKV ≥ 1500 mL | 3.08 (1.71–5.55) | 0.0002* | 4.38 (1.15–16.71) | 0.0304* |
|  |  |  |  |  |
| htTKV < 500 mL | 1 (reference) |  | 1 (reference) |  |
| htTKV 500–1000 mL | 2.13 (1.08–4.19) | 0.0293* | 1.77 (0.41–7.63) | 0.4444 |
| htTKV ≥ 1000 mL | 3.61 (1.85–7.06) | 0.0002* | 3.41 (0.86–13.50) | 0.0807 |
|  |  |  |  |  |
| Mayo class 1A | 1 (reference) |  | 1 (reference) |  |
| Mayo classes 1B–1C | 1.23 (0.72–2.11) | 0.4385 | 1.27 (0.41–3.94) | 0.6845 |
| Mayo classes 1D–1E | 2.70 (1.48–4.92) | 0.0012* | 5.65 (1.63–19.54) | 0.0063* |
|  |  |  |  |  |
| CKD stages 1–2 | 1 (reference) |  | 1 (reference) |  |
| CKD stage 3 | 1.65 (0.83–3.26) | 0.1541 | 2.34 (0.54–10.14) | 0.2545 |
| CKD stages 4–5 | 3.99 (2.04–7.80) | <0.0001* | 4.91 (1.20–20.16) | 0.0272* |

**P* < 0.05. Abbreviations: IA, intracranial aneurysm; SAH, subarachnoid hemorrhage; ADPKD, autosomal dominant polycystic kidney disease; TKV, total kidney volume; htTKV, height-adjusted total kidney volume; Mayo 1D–1E, Mayo imaging classification 1D–1E; CKD, chronic kidney disease; CI, confidence interval; *P*, calculated probability.

**S3 Table. Multivariable logistic regression analyses for correlations between a diagnosis of intracranial aneurysms and risk factors**

|  | **General Risk Factors**  **With htTKV (*n* = 436)** |  | **General Risk Factors**  **With Mayo 1D–1E (*n* = 429)** |  |
| --- | --- | --- | --- | --- |
|  | (AICc = 377.5, pseudo-*R*^2^=0.11, AUC=0.72) |  | (AICc = 382.7, pseudo-*R*^2^=0.10, AUC=0.71) |  |
| **Variables** | Odds Ratio (95% CI) | *P*-Value | Odds Ratio (95% CI) | *P*-Value |
| Female (vs. male) | 1.96 (1.10–3.51) | 0.0228* | 1.69 (0.96–2.97) | 0.0688 |
| Hypertension | 2.06 (1.10–3.84) | 0.0237* | 1.99 (1.07–3.71) | 0.0294* |
| Family history of IA or SAH | 2.87 (1.57–5.27) | 0.0007* | 2.71 (1.49–4.93) | 0.0011* |
| Age (10-year increments) | 1.13 (0.92–1.39) | 0.2412 | 1.42 (1.12–1.79) | 0.0031* |
| htTKV (100 mL increase) | 1.09 (1.05–1.14) | <0.0001* | NA | NA |
| Mayo 1D–1E | NA | NA | 2.90 (1.52–5.52) | 0.0012* |
|  | **General Risk Factors**  **With htTKV ≥500 mL (*n* = 436)** |  | **General Risk Factors**  **With Mayo 1D–1E (*n* = 429)** |  |
|  | (AICc = 387.0, pseudo-*R*^2^=0.09, AUC=0.71) |  | (AICc = 386.4, pseudo-*R*^2^=0.09, AUC=0.70) |  |
| **Variables** | Odds Ratio (95% CI) | *P*-Value | Odds Ratio (95% CI) | *P*-Value |
| Female (vs. male) | 1.61 (0.93–2.79) | 0.0877 | 1.65 (0.94–2.88) | 0.0811 |
| Hypertension | 2.20 (1.20–4.03) | 0.0111* | 2.34 (1.28–4.27) | 0.0056* |
| Family history of IA or SAH | 2.98 (1.64–5.40) | 0.0003* | 2.81 (1.56–5.10) | 0.0006* |
| Age ≥ 50 years | 1.27 (0.74–2.18) | 0.3820 | 1.99 (1.10–3.62) | 0.0239* |
| htTKV ≥500 mL | 2.81 (1.47–5.39) | 0.0018* | NA | NA |
| Mayo 1D–1E | NA | NA | 2.52 (1.35–4.71) | 0.0037* |

**P* < 0.05. Variables of general risk factors for intracranial aneurysms, htTKV, and Mayo 1D–1E were included in the multivariable models. Abbreviations: n, number; htTKV, height-adjusted total kidney volume; Mayo 1D–1E, Mayo imaging classification 1D–1E; CI, confidence interval; *P*, calculated probability; AICc, small-sample corrected Akaike Information Criterion; pseudo-*R*^2^, McFadden’s pseudo-*R*-squared; AUC, area under the receiver operating characteristic curve; IA, intracranial aneurysm; SAH, subarachnoid hemorrhage; NA, not applicable

**S1 Figure. Odds ratios for a diagnosis of intracranial aneurysm from multivariable logistic regression analyses (A–D)**


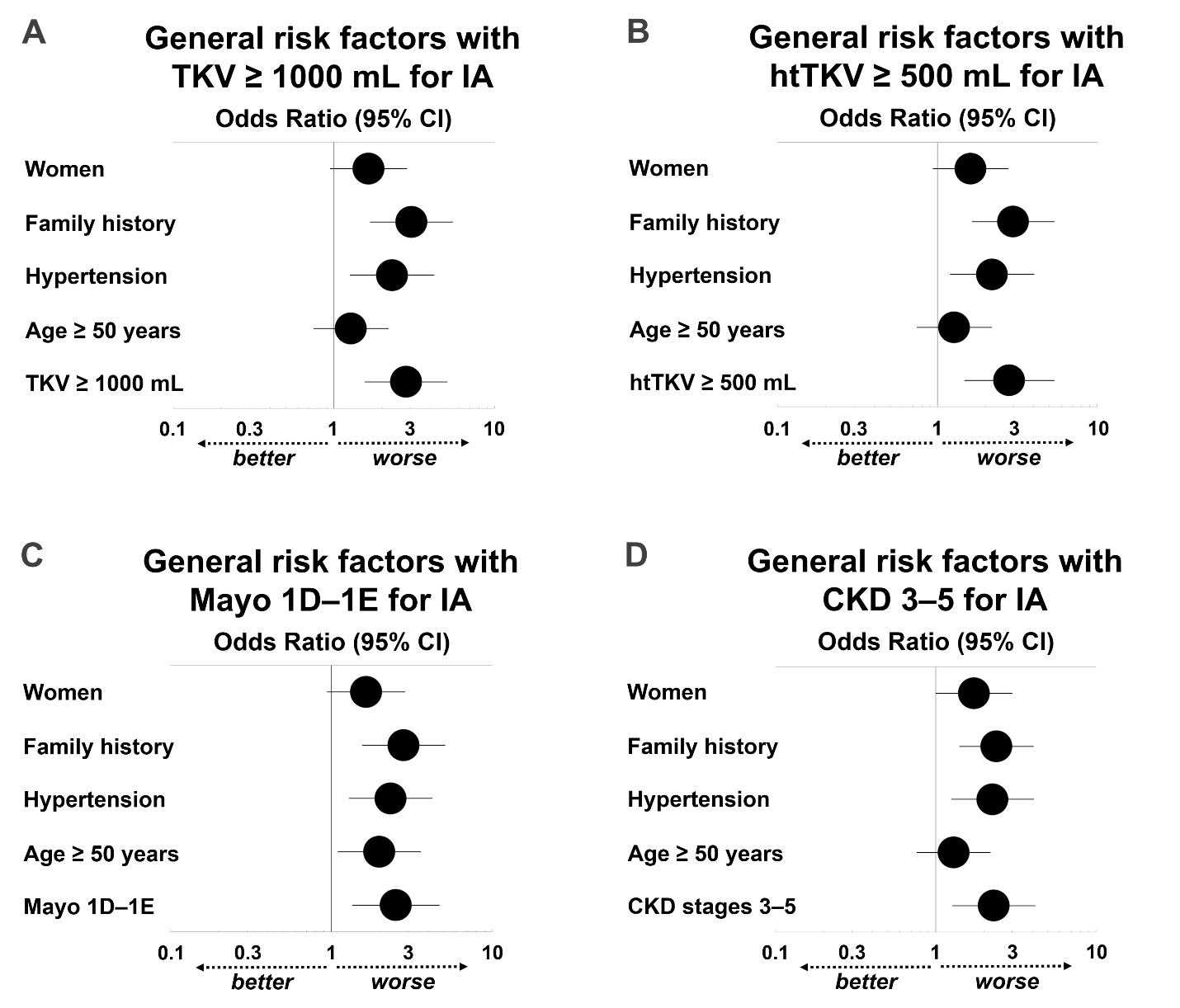


The circles represent odds ratios, and the bars represent 95% CI for the association with IA diagnosis (Table 3, lower part). (A) General risk factors with TKV ≥1000 mL for IA. (B) General risk factors with htTKV ≥500 mL for IA. (C) General risk factors with Mayo 1D–1E. (D) General risk factors with CKD stages 3–5. Abbreviations: IA, intracranial aneurysm; CI, confidence interval; TKV, total kidney volume; htTKV, height-adjusted total kidney volume; Mayo 1D–1E, Mayo imaging classification 1D–1E; CKD, chronic kidney disease
